# 5‐aminolevulinic acid photodynamic therapy protects against UVB‐induced skin photoaging: A DNA‐repairing mechanism involving the BER signalling pathway

**DOI:** 10.1111/jcmm.18536

**Published:** 2024-07-23

**Authors:** Jing Wang, Li Gu, Zhinan Shi, Zhiyi Xu, Xiaoyu Zhai, Shu Zhou, Jingting Zhao, Liqun Gu, Lin Chen, Linling Ju, Bingrong Zhou, Hui Hua

**Affiliations:** ^1^ Department of Dermatology, Nantong Third People's Hospital Affiliated Nantong Hospital 3 of Nantong University Nantong China; ^2^ Medical School of Nantong University Nantong China; ^3^ Nantong Institute of Liver Diseases, Nantong Third People's Hospital Affiliated Nantong Hospital 3 of Nantong University Nantong China; ^4^ Department of Dermatology The First Affiliated Hospital of Nanjing Medical University Nanjing China

**Keywords:** BER pathway, DNA damage, photoaging, photodynamic therapy, RNA‐seq, UVB

## Abstract

Low‐dose 5‐aminolevulinic acid photodynamic therapy (ALA‐PDT) has been used to cope with skin photoaging, and is thought to involve DNA damage repair responses. However, it is still unknown how low‐dose ALA‐PDT regulates DNA damage repair to curb skin photoaging. We established a photoaging model using human dermal fibroblasts (HDFs) and rat skin. RNA‐sequencing (RNA‐seq) analysis was conducted to identify differentially expressed genes (DEGs) in HDFs before and after low‐dose ALA‐PDT treatment, followed by bioinformatics analysis. Senescence‐associated β‐galactosidase (SA‐β‐gal) staining was employed to assess skin aging‐related manifestations and Western blotting to evaluate the expression of associated proteins. A comet assay was used to detect cellular DNA damage, while immunofluorescence to examine the expression of 8‐hydroxy‐2′‐deoxyguanosine (8‐oxo‐dG) in cells and skin tissues. In both in vivo and in vitro models, low‐dose ALA‐PDT alleviated the manifestations of ultraviolet B (UVB)‐induced skin photoaging. Low‐dose ALA‐PDT significantly reduced DNA damage in photoaged HDFs. Furthermore, low‐dose ALA‐PDT accelerated the clearance of the photoproduct 8‐oxo‐dG in photoaged HDFs and superficial dermis of photoaged rat skin. RNA‐seq analysis suggested that low‐dose ALA‐PDT upregulated the expression of key genes in the base excision repair (BER) pathway. Further functional validation showed that inhibition on BER expression by using UPF1069 significantly suppressed SA‐β‐gal activity, G2/M phase ratio, expression of aging‐associated proteins P16, P21, P53, and MUTYH proteins, as well as clearance of the photoproduct 8‐oxo‐dG in photoaged HDFs. Low‐dose ALA‐PDT exerts anti‐photoaging effects by activating the BER signalling pathway.

## 1 INTRODUCTION

Skin photoaging, usually caused by ultraviolet (UV) radiation from sunlight,^1^ is closely associated with various age‐related skin diseases, even benign and malignant skin tumours.^2^ Current main treatments include chemical peels, laser therapy, botulinum toxin injections and microneedle radiofrequency. More safe and effective options should be explored for the prevention and treatment of skin photoaging.

5‐aminolevulinic acid photodynamic therapy (ALA‐PDT) employs photosensitizers to irradiate target cells with specific wavelengths of light, thus generating reactive oxygen species (ROS) that promote oxidative stress to achieve therapeutic purposes.^3^ Unlike high‐dose ALA‐PDT that induces high levels of ROS resulting in cell apoptosis or even necrosis, low‐dose ALA‐PDT just causes minimal oxidative damage to cells, but meanwhile enhances cell proliferation, skin healing, and endogenous defence.^4^, ^5^ Numerous clinical studies have shown beneficial effects of ALA‐PDT on various symptoms of skin photoaging. Zhang et al. demonstrated that multiple sessions of low‐dose ALA‐PDT could reduce wrinkles and pigmentation in the long term.^6^ Additionally, several studies identified the anti‐photoaging mechanisms of low‐dose ALA‐PDT. Chen, J. et al. found that low‐dose ALA‐PDT could activate Nrf2 in photoaged cells and produce antioxidant stress responses to achieve therapeutic effects on photoaging in cellular experiments.^3^ Another study revealed that low‐dose ALA‐PDT inhibited the senescence of photoaged fibroblasts through upregulation on Bach2.^4^ Although these studies suggest that low‐dose ALA‐PDT ameliorates photoaging by enhancing the activity of photoaged fibroblasts, but the exact mechanisms of action is still far from transparency.

Skin aging involves the decline of DNA‐repairing capacity and accumulation of DNA photoproducts following exposure to UV radiation.^7^, ^8^ Generated upon UV radiation, the ROS can indirectly damage DNA by inducing the oxidation of 8‐oxo‐dG and this damage is primarily repaired by the BER system.^9^, ^10^ Increasing evidence suggests that DNA damage repair plays a crucial role in the process of photoaging. Clinical studies found that if added with DNA‐repairing enzymes (photolyase and T4 endonuclease V), conventional sunscreen showed a stronger capacity to repair DNA and inhibit cell apoptosis, thus assisting in preventing photoaging.^11^ Our previous research also revealed that low‐dose ALA‐PDT significantly increased the expression of proteins in DNA damage repair pathways and accelerated the clearance of DNA photoproducts induced by UV radiation in HDFs.^12^ However, the precise mechanism by which low‐dose ALA‐PDT regulates the DNA damage repair pathways remains unknown.

In this study, we established UVB‐induced cellular and animal models of skin photoaging to illustrate how low‐dose ALA‐PDT regulates the DNA damage repair pathways.

## 2 METHODS

### 2.1 Cell culture

HDFs were obtained from the foreskin of healthy male patients at the Department of Urology, Nantong Third People's Hospital. This study had obtained approval from the Medical Ethics Committee of Nantong Third People's Hospital (No: EL2022007). The foreskin was washed repeatedly with phosphate buffered saline (PBS) for three times, incubated with 0.5% Dispase enzyme at 4°C overnight, and then digested with collagenase for 1 h at 37°C. The suspension was filtered and centrifuged, and the supernatant was discarded. Cell fragments were collected and cultured in Dulbecco's modified Eagle's medium (DMEM, Thermo Fisher Scientific) supplemented with 10% foetal bovine serum (Every Green, Zhejiang Tianhang Biotechnology Co., Ltd.) and 1% penicillin–streptomycin (New Cell & Molecular Biotech Co. Ltd.) at 37°C in a 5% CO_2_ incubator. Experiments were conducted using logarithmic growth phase fibroblasts from passages 4–10.

### 2.2 HDFs photoaging model

HDFs were inoculated into culture dishes and allowed to grow to 80% confluency. Then, they were washed with PBS and sufficient PBS was added to completely cover the cells. Subsequently, HDFs were exposed to UVB radiation from a UVB fluorescent lamp (JCB35‐24‐01, Sigma, Shanghai, China) at a distance of 15 cm. The radiation intensity of the UVB lamp was calibrated using a UVB radiation meter (Sigma, Shanghai, China). The cells were irradiated twice a day for five consecutive days, with a daily dose of 10 mJ/cm^2^. After irradiation, the PBS was removed and an appropriate amount of DMEM culture medium was added for further cultivation.

### 2.3 ALA‐PDT treatment

Cells were randomly divided into three groups: Control group, UVB‐SIPS group (10 J/cm^2^ UVB irradiation for 5 days), and UVB‐SIPS+ALA‐PDT group (10 J/cm^2^ UVB irradiation for 5 days followed by 0.5 mmol/L ALA treatment and 6 J/cm^2^ red light irradiation). In the UVB‐SIPS+ALA‐PDT group, the cells were incubated with serum‐free DMEM medium containing 0.5 mmol/L ALA (Shanghai Fudan‐zhangjiang Bio‐Pharmaceutical Co., Ltd) in the dark for 1.5 h, followed by red light irradiation (635 ± 5 nm, 6 J/cm^2^, Shenzhen Lifotronic Technology Co., Ltd.) at a distance of 15 cm from the cells. After light exposure, the original culture medium was discarded, and the cells were washed three times with PBS. Then, an appropriate amount of DMEM culture medium was added and the cells were further cultured in an incubator at 37°C, 5% CO_2_, and saturated humidity.

### 2.4 Senescence‐associated β‐galactosidases (SA‐β‐gal) staining

The SA‐β‐gal activity was detected using the SA‐β‐gal staining kit (Beyotime Biotechnology). Cells were washed with PBS once and then incubated with 1 mL of SA‐β‐gal staining fixative at room temperature for 15 min. After washing with PBS three times, 1 mL of staining working solution was added and the cells were incubated overnight at 37°C. The staining results were observed under an optical microscope, and the senescent cells were identified by the intense blue cytoplasmic staining. At least 400 cells were counted to quantify the senescence of cells.

### 2.5 Cell cycle analysis

Cell cycle analysis was performed using the Cell Cycle Detection Kit (KGA511, Keygen, China). Cells from each group were collected, washed with PBS twice, treated with 70% pre‐chilled ethanol, and stored at −20°C overnight. After removing the ethanol by washing with PBS twice, the cells were stained with 50 mg/L propidium iodide and 50 mg/L RNAase, and incubated at room temperature for 30 min in the dark. The cell cycle was analysed using a flow cytometer (FAC‐Scan, BD, NJ, USA).

### 2.6 Modified alkaline comet assay

The OxiSelect™ Comet Assay Kit (Cell Biolabs, Inc., San Diego, CA) was used to assess DNA damage. Cells were mixed with comet agarose, allowed to solidify at 4°C, treated with pre‐chilled lysis buffer for 60 min, followed by incubation with formamidopyrimidine DNA glycosylase (Fpg) (New England Biolabs, Beverly, MA, USA) for 30 min, and then treatment with alkaline solution for another 30 min. Subsequently, the samples were transferred to a horizontal electrophoresis chamber with TBE buffer and electrophoresed at 25 V for 15 min. After staining with Vista Green DNA dye for 15 min, the slides were observed using a fluorescent microscope with FITC filters. Every 50 cells were randomly selected, and the comet images were analysed using CASP software. The percentage of Tail DNA was measured to quantify DNA damage.

### 2.7 Quantitative reverse transcription polymerase chain reaction (qRT‐PCR)

Total RNA was extracted using TRIzol reagent (Takara, Shiga, Japan) and cDNA was synthesized using the PrimeScript RT Reagent Kit (Takara, Shiga, Japan). qRT‐PCR was performed using the TB Green Premix Ex Taq II (Takara, Shiga, Japan) on the BIO‐RAD CFX system (BioRad, Munich, Germany). Each sample was replicated three times. The relative mRNA expression levels were calculated using the 2^−ΔΔCT^ method with GAPDH as a reference gene. The primers were designed by Sangon Biotech (Shanghai) Co., Ltd., and the primer sequences used in this experiment can be found in the Supplementary Materials.

### 2.8 Western blot analysis

Total cellular proteins were extracted using RIPA lysis buffer (Biyuntian, Beijing, China). The protein concentration was measured using the BCA Protein Assay Kit (Beyotime Biotechnology, China). The proteins were separated by SDS‐PAGE and transferred onto a PVDF membrane. The membrane was then blocked with TBST buffer (0.05% Tween 20 in TBS buffer) containing 5% bovine serum albumin (BSA). Primary antibodies against P16, P21, P53, MUTYH and β‐actin (Abcam, Cambridge, UK) were incubated overnight at 4°C, followed by washing. The membrane was then incubated with goat anti‐rabbit HRP secondary antibody (Abcam, Cambridge, UK) for 1 h. The protein bands were visualized using a gel imaging system (Tanon 5200 Multi, Shanghai, China), and quantified using ImageJ open‐source software (version 1.53e). The protein expression was normalized to β‐actin.

### 2.9 Immunofluorence

Cells were cultured on coverslips and fixed with 4% paraformaldehyde solution at room temperature for 15 min. After washing three times with PBS, the cells were permeabilized with 0.5% Triton X‐100 for 15 min. Subsequently, the cells were blocked with 5% BSA at 37°C for 30 min. Mouse anti‐8‐oxo‐dG antibody (1:100, Santa Cruz, USA) was then applied and the cells were incubated overnight at 4°C. The next day, Alexa Flour 488 and CY3 fluorescent secondary antibodies (1:400) were added and the cells were incubated at 37°C for 1 h, followed by three times of washing with PBS. The cell nuclei were stained with DAPI (Sigma, USA) for 5 min. Immunofluorescence was detected using a confocal microscope to determine the percentage of cells positive for 8‐oxo‐dG.

### 2.10 RNA sequencing (RNA‐Seq) analysis

Cells from the Control group, UVB‐SIPS group, and UVB‐SIPS+ALA‐PDT group were collected using TRIzol reagent (Takara, Shiga, Japan). Three cell samples were collected for each group. The RNA was then isolated and purified according to the manufacturer's protocol. RNA‐Seq was performed in Nanjing Novogene Bioinformatics Technology Co., Ltd. After data processing using HISAT2 software, we determined *p*_adj_ <0.05 as a criterion for differentially expressed genes (DEGs). Gene Ontology (GO) and Kyoto Encyclopedia of Genes and Genomes (KEGG) analyses were conducted using the David database (https://david.ncifcrf.gov/). Visualization and plotting were performed using R (R 4.2.0).

### 2.11 BER inhibitor UPF1069 treatment

A 10% DMEM solution containing 10 mM of UPF1069 (HY‐14478, MCE) was prepared, added to a culture dish, and incubated at 37°C for 2 h. The culture medium was removed, and the dish was washed twice with PBS buffer. All residual liquid was ensured to be removed. Then, fresh 10% DMEM medium was added to continue the cultivation.

### 2.12 Animals

Male SD rats (6–8 weeks old, weighing 209 ± 15 g) were provided by the Experimental Animal Centre of Nantong University. All animal experiments were approved by the Animal Ethics Committee of Nantong University (No: S20231128‐099) and conducted in accordance with their guidelines. After 1 week of acclimation, the dorsal skin of the rats was exposed by approximately 5 cm × 5 cm in size, and subjected to shaving and depilation. The rats were then randomly divided into three groups, with three rats in each group: Control group (no treatment), UVB group (6.9 J/cm^2^ UVB), and UVB + ALA‐PDT group (6.9 J/cm^2^ UVB + 3% ALA +6 J/cm^2^ PDT).

### 2.13 Rat skin photoaging model and ALA‐PDT treatment

UVB irradiation was performed using UVB fluorescent lamps (JCB35‐24‐01, Sigma, Shanghai, China). UVB irradiance was measured using a UV radiation intensity meter (Sigma, Shanghai, China). SD rats were placed in a homemade irradiation box (with their eyes shielded) and positioned 10 cm below the UVB light source. The rats were exposed to UVB light 5 days a week for 8 weeks. The dosing for UVB irradiation was as follows: 60 mJ/cm^2^ for the first and second weeks, 120 mJ/cm^2^ for the third week, 180 mJ/cm^2^ for the fourth week, and 240 mJ/cm^2^ for the fifth to eighth weeks, till a cumulative dose of 6.9 J/cm^2^. After 8 weeks, UVB irradiation was discontinued, and ALA‐PDT treatment was initiated. For the UVB + ALA‐PDT group, a 3% ALA cream was evenly applied to the dorsal area of each rat. Multiple layers of plastic film and gauze were then applied, followed by fixation using medical tape. After 3 h of incubation in a dark room, excess ALA cream was removed, and irradiation was performed with red light (Shenzhen Lifotronic Technology Co., Ltd.) at a wavelength of 635 ± 5 nm, with a total light dose of 6 J/cm^2^ per rat. The frequency was set at once a week, for a total of three times.

### 2.14 Dermoscopy and histopathological examination

After rats were anaesthetised, a hand‐held dermatoscope was used to take pictures of the skin on the back of each rat. After 4 weeks of ALA‐PDT treatment, the rats in each group were euthanized, and dorsal skin tissue was collected and fixed in 4% paraformaldehyde solution. The tissue specimens were then embedded in paraffin and sectioned. Haematoxylin and eosin (H&E) staining was performed to examine histological features and measure skin thickness. Masson trichrome staining and Elastica van Gieson (EVG) staining were employed to evaluate collagen fibres and elastic fibres, respectively. Image‐Pro Plus software (Media Cybernetics) was used for analysis.

### 2.15 Immunofluorescence analysis on 8‐oxo‐dG

All specimens were fixed with 4% paraformaldehyde and embedded in paraffin. Blocking was performed with BSA for 30 min, followed by overnight incubation at 4°C with mouse anti‐8‐oxo‐dG antibody (1:100, Santa Cruz, USA). The specimens were then incubated for 1 h at 37°C with CY3‐labelled goat anti‐mouse secondary antibody (1:300, Servicebio, China). Subsequently, the specimens were observed and photographed under a fluorescence microscope, to compare the percentage of 8‐oxo‐dG positive cells.

### 2.16 Statistics

Statistical analysis was performed using GraphPad Prism 8 (GraphPad Software Inc., San Diego, CA, USA). The statistical analysis was carried out using the *t*‐test or the one‐way analysis of variance (ANOVA). All normally distributed data were expressed as standard deviation ± SD. *p* < 0.05 was considered statistically significant.

## 3 RESULTS

### 3.1 Low‐dose ALA‐PDT inhibited UVB‐induced photoaging in HDFs

**FIGURE 1:**
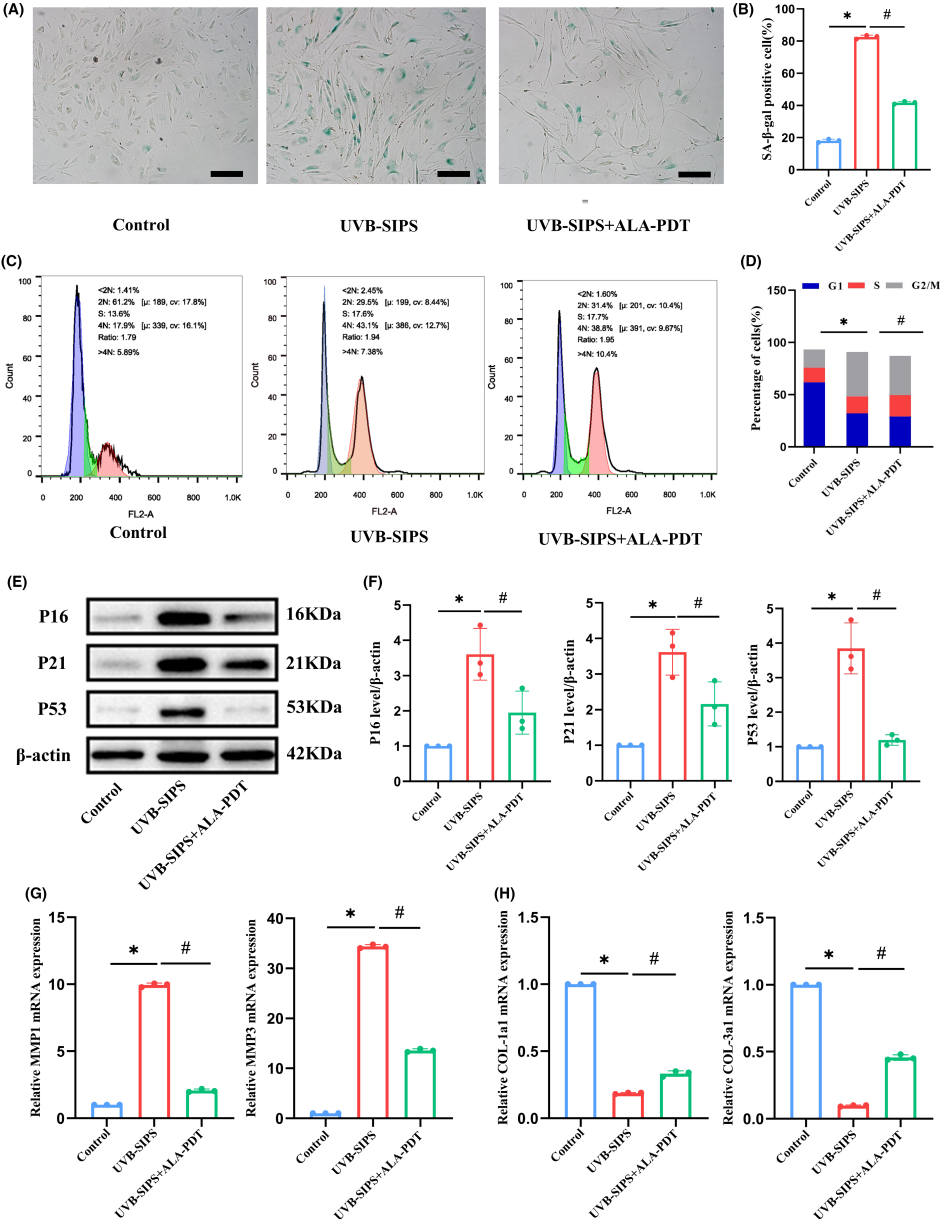
Low‐dose ALA‐PDT inhibited UVB‐induced photoaging in HDFs. (A) SA‐β‐gal staining. Bar = 600 μm, *N* = 3. (B) Quantitative analysis of SA‐β‐gal staining positive cells. (C) Cell cycle analysis of HDFs in each group was performed using flow cytometry. (D) The percentage of cells in each phase of the cell cycle. (E) The levels of P16, P21, and P53 proteins were detected by Western blot in each group of HDFs. (F) The bar graph represents the ratio of the grey values of the target protein/β‐Actin. (G) MMP‐1 and MMP3 mRNA in each group of HDFs were detected by qRT‐PCR. (H) COL1a1, COL3a1 mRNA of HDFs in each group were detected by qRT‐PCR. *p < 0.05 vs. Control group, #*p* < 0.05 vs. UVB‐SIPS group, *N* = 3.

Compared to the control group, the UVB‐SIPS group exhibited a larger volume of HDFs with a flattened morphology. In contrast, the UVB‐SIPS+ALA‐PDT group showed lower cell density and volume (Figure 1A). The percentage of SA‐β‐gal positive cells in the UVB‐SIPS group was 82.57 ± 1.14%, whereas 41.76 ± 0.81% in the UVB‐SIPS+ALA‐PDT group (*p* < 0.05) (Figure 1B). Flow cytometry was used to detect the cell cycles among groups. As shown in Figure 1C,D, the proportion of G2/M phase cells in the UVB‐SIPS group was 42.50 ± 1.22%, but decreased to 36.97 ± 1.68% in the UVB‐SIPS+ALA‐PDT group (*p* < 0.05). Western blot results (Figure 1E,F) demonstrated a significant decrease in the protein expression levels of P16, P21 and P53 in the UVB‐SIPS+ALA‐PDT group compared to the UVB‐SIPS group (*p* < 0.05). qRT‐PCR results (Figure 1G,H) showed a significant reduction in MMP‐1 and MMP‐3 mRNA expression and a significant increase in COL1a1 and COL3a1 mRNA expression in the UVB‐SIPS+ALA‐PDT group compared to the UVB‐SIPS group (*p* < 0.05).

### 3.2 Low‐dose ALA‐PDT inhibited UVB‐induced photoaging in rat skin

**FIGURE 2:**
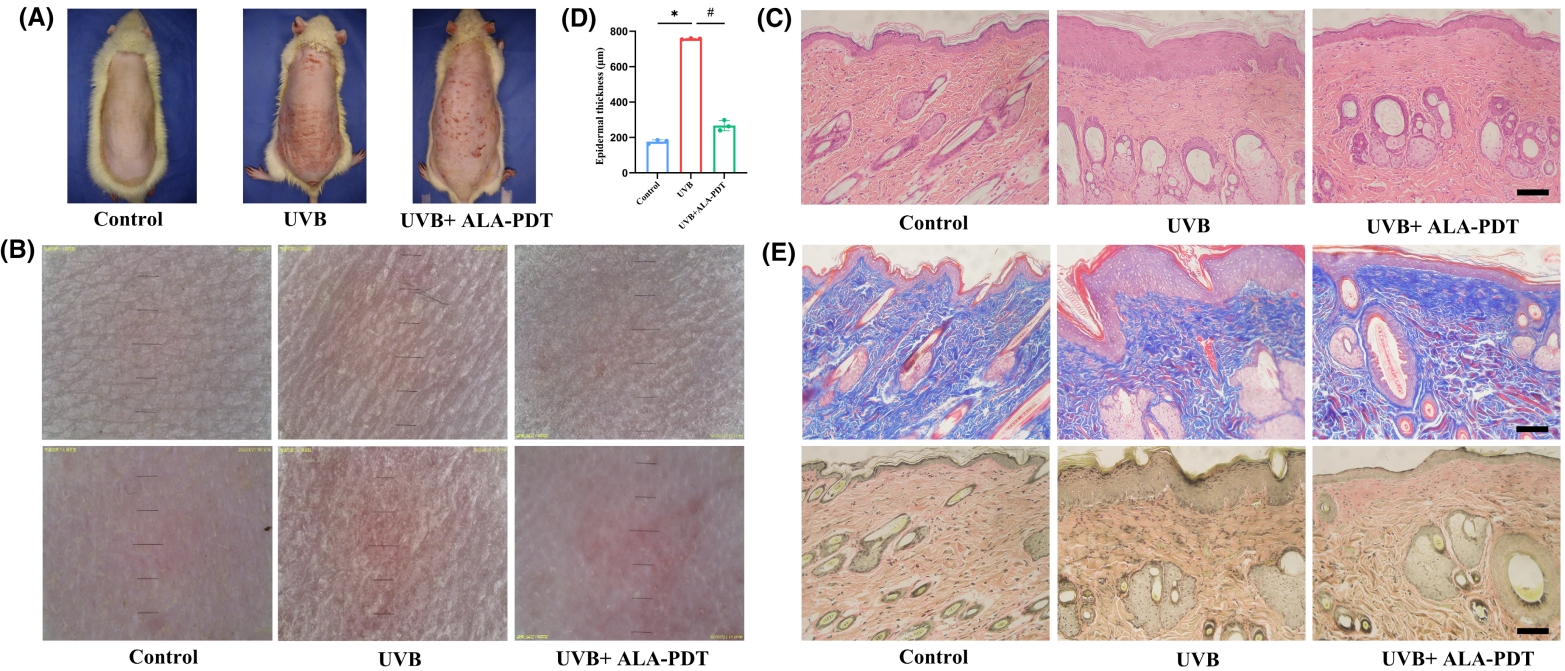
Low‐dose ALA‐PDT inhibited UVB‐induced skin photoaging in rats. (A) The appearance of rats in Control, UVB, and UVB + ALA‐PDT groups is shown in representative photographs. (B) The micro appearance of rats shown by dermoscopic images. (C) The histological changes in epidermis and dermis were observed by H&E staining. (D) Epidermal thickness. (E) Histological changes in collagen and elastic fibres observed by Masson staining and EVG staining. *p < 0.05 vs. Control group, # *p* < 0.05 vs. UVB group, Bar =500 μm, N = 3.

After chronic UVB irradiation, rats exhibited typical signs of photoaging, including dry and rough skin with a leathery appearance and deep wrinkles. Following low‐dose ALA‐PDT treatment, the skin became smoother, and the depth of wrinkles decreased (Figure 2A). Dermoscopy revealed that compared to the control group, the UVB group showed widened skin furrows with an uneven distribution of red patches. After low‐dose ALA‐PDT treatment, the skin condition significantly improved with a reduction in redness and finer texture (Figure 2B). H&E staining (Figure 2C,D) demonstrated that the epidermal thickness in the ALA‐PDT group was 267.0 ± 29.21 μm, significantly lower than the 757.30 ± 3.06 μm in the UVB group (*p* < 0.05). Masson and EVG staining showed that, compared to the control group, the UVB group exhibited a decrease in collagen fibre content in the dermis with a disorganized and irregular arrangement. Elastic fibres appeared thickened, distorted, fragmented, and partially degraded into amorphous masses. After low‐dose ALA‐PDT treatment, collagen fibres significantly increased, exhibiting a more orderly and denser arrangement. Elastic fibres appeared slender, well‐organized and partially interwoven (Figure 2E).

### 3.3 Low‐dose ALA‐PDT reduced UVB‐induced DNA damage

**FIGURE 3:**
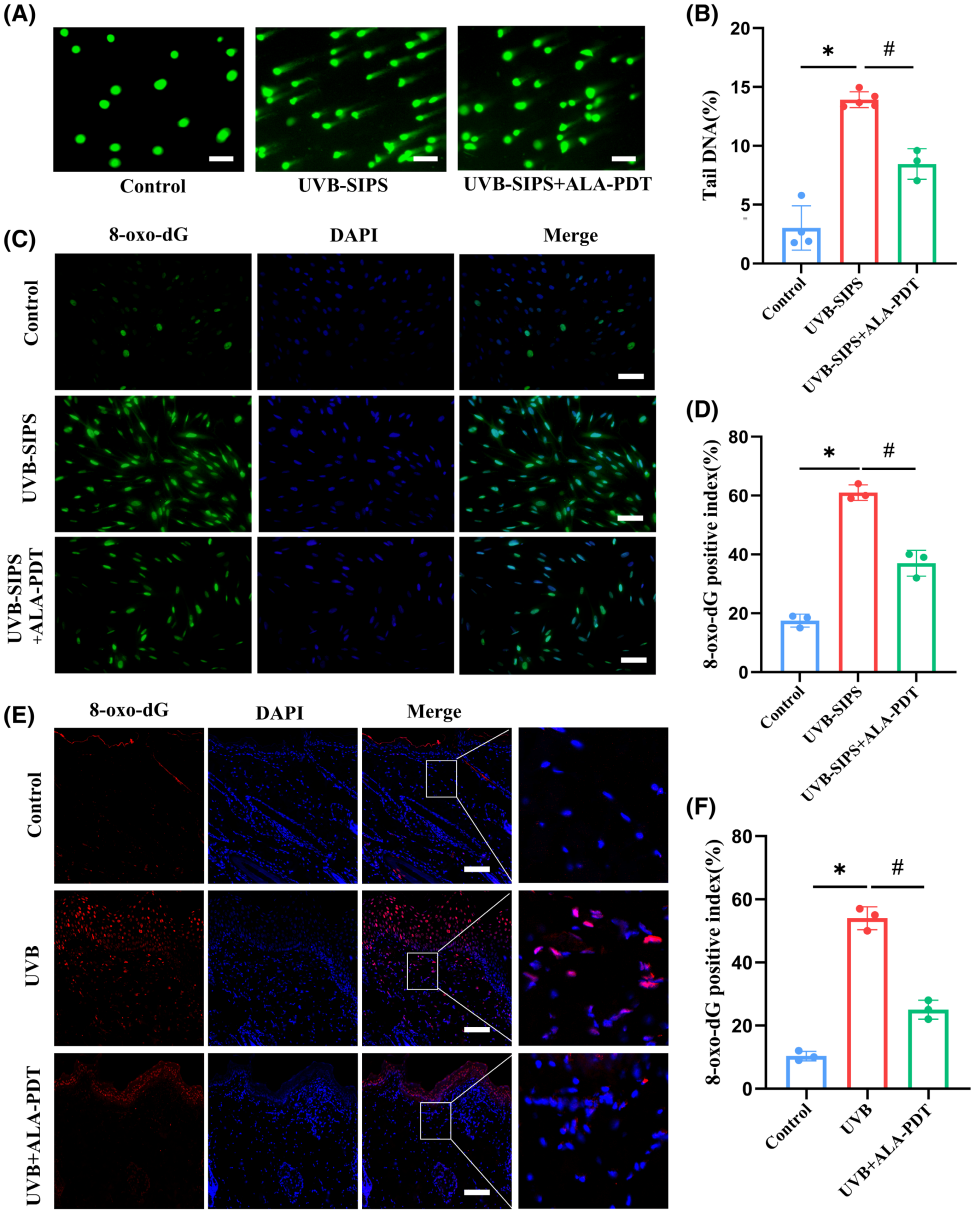
Low‐dose ALA‐PDT reduced DNA damage in UVB‐ photoaged HDFs and rat dorsal skin. (A) DNA damage was detected using a comet assay. Representative comet assay images were captured under a fluorescent microscope. Bar = 200 μm, *N* = 3. (B) The bar graph represents the percentage of tail DNA. (C) The detection of 8‐oxo‐dG‐positive cells in each group of HDFs by immunofluorescence assay. Bar = 400 μm, *N* = 3. (D) Quantitative analysis of 8‐oxo‐dG‐positive cells in each group of cells. (E) The detection of 8‐oxo‐dG‐positive cells in epidermis and superficial dermis of each group by an immunofluorescence assay. Bar = 100 μm, *N* = 3. (F) Quantitative analysis of 8‐oxo‐dG‐positive cells in the superficial dermis of various groups. *p < 0.05 vs. Control group, #*p* < 0.05 vs. UVB‐SIPS group, #*p* < 0.05 vs. UVB group, *N* = 3.

The results of the comet assay (Figure 3A,B) showed that the tail DNA content in the UVB‐SIPS group was 13.92 ± 0.67%, whereas after treatment with ALA‐PDT, the tail DNA content in photoaged HDFs decreased to 8.46 ± 1.30% (*p* < 0.05). Immunofluorescence staining for 8‐oxo‐dG revealed that in the UVB‐SIPS group, the proportion of 8‐oxo‐dG‐positive cells was 61.0 ± 2.65%. However, after treatment with ALA‐PDT in photoaged HDFs, the proportion of 8‐oxo‐dG‐positive cells decreased to 37.0 ± 4.36% (*p* < 0.05) (Figure 3C,D). In vivo, we also observed the presence of 8‐oxo‐dG in the epidermis and superficial dermis of UVB‐induced photoaged rats. The proportion of 8‐oxo‐dG‐positive cells in the superficial dermis of photoaged rats was 54.0 ± 3.61%. However, after treatment with low‐dose ALA‐PDT, the proportion of 8‐oxo‐dG‐positive cells in the superficial dermis of photoaged rats decreased to 25.0 ± 3.0% (*p* < 0.05) (Figure 3E,F).

### 3.4 mRNA expression profile of DEGs after low‐dose ALA‐PDT treatment in photoaged HDFs

**FIGURE 4:**
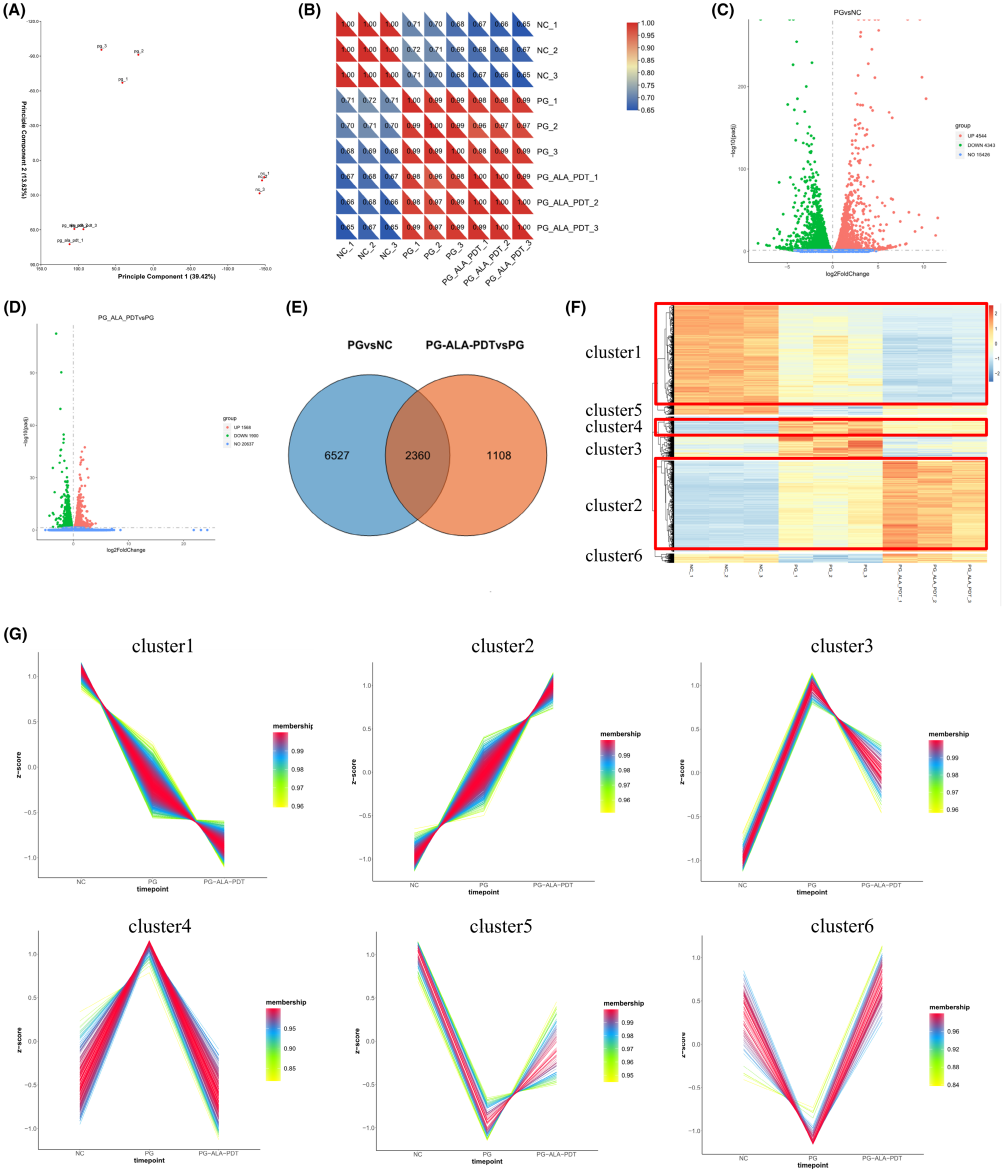
Analysis of DEGs following low‐dose ALA‐PDT treatment in UVB‐induced photoaged HDFs. (A) Scatter plot of PCA analysis. (B) Unsupervised clustering correlation heatmap. (C, D) The volcano diagram indicates all genes, green dots indicate down‐regulated genes, and red dots indicate up‐regulated genes. (E) Venn diagram showing the intersection DEGs between UVB‐SIPS vs control and UVB‐SIPS+ALA‐PDT vs UVB‐SIPS. (F, G) The cluster analysis revealed six clusters of DEGs and showed their expression trends. Red lines correspond to genes with high affiliation. Each square represents a profile of a gene expression trend. PCA, Principal Component Analysis; NC, Control group; PG, UVB‐SIPS group; PG‐ALA‐PDT, UVB‐SIPS+ALA‐PDT group.

RNA‐seq was performed on cells collected from the Control group, UVB‐SIPS group, and UVB‐SIPS+ALA‐PDT group. The correlation heatmap and principal component analysis (PCA) indicated consistency within each group and differences between groups (Figure 4A,B). A total of 8887 DEGs (*p*_adj_ <0.05) were identified between the UVB‐SIPS group and the Control group, with 4544 upregulated and 4343 downregulated genes. When comparing the UVB‐SIPS+ALA‐PDT group to the UVB‐SIPS group, 3468 DEGs (*p*_adj_ <0.05) were determined, with 1568 upregulated and 1900 downregulated (Figure 4C,D). The Venn diagram showed 2360 common DEGs obtained from the intersections between the two groups (Figure 4E). Using the R package Mfuzz, the differential gene clustering heatmap was generated, demonstrating 6 obvious clusters (Figure 4F). Cluster 1 showed a decreasing trend, cluster 2 an increasing trend, clusters 3 and 4 an initial increase followed by a decrease trend, and clusters 5 and 6 an initial decrease followed by an increase trend. Among these clusters, genes in clusters 3, 4, 5, and 6 exhibited expression changes induced by UVB that could be counteracted by ALA‐PDT (Figure 4G).

### 3.5 GO/KEGG enrichment analysis of key DEGs

**FIGURE 5:**
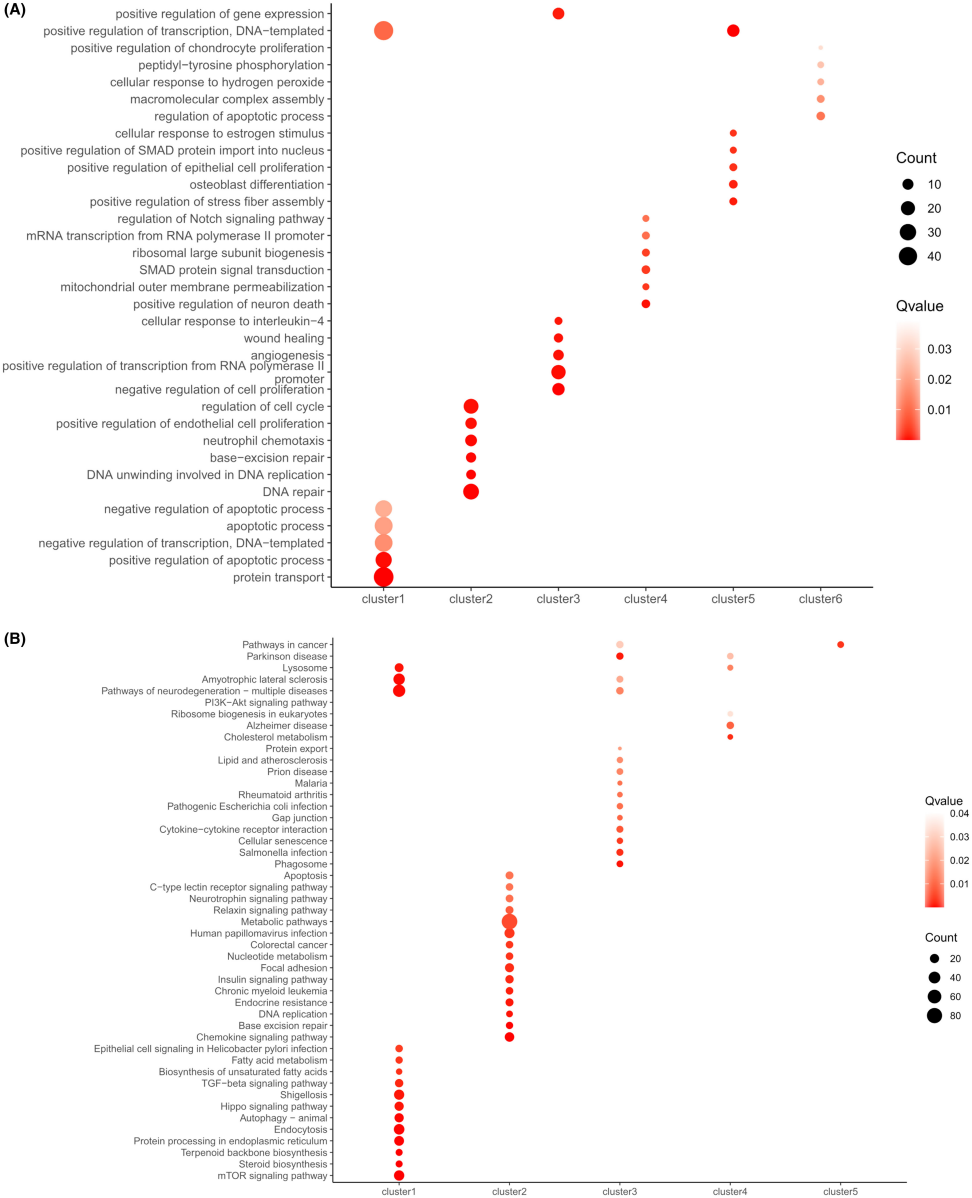
GO/KEGG enrichment analysis of HDFs after low‐dose ALA‐PDT treatment for improvement of UVB‐induced photoaging. (A) GO enrichment analysis of six clusters. (B) KEGG enrichment analysis of six clusters. Each GO term is represented by a bubble. The colour intensity of each bubble indicates the p adjust value of the corresponding GO term, with a size corresponding to the proportion of query genes in the gene set associated with the given GO term. GO, Gene Ontology; KEGG, Kyoto Encyclopedia of Genes and Genomes. Three groups: Control group, UVB‐SIPS group, UVB‐SIPS+ALA‐PDT group.

As shown in GO enrichment analysis (Figure 5A), Cluster 1 was mainly associated with the regulation of cellular autophagy, apoptosis, and cholesterol biosynthesis. Cluster 2 was related to DNA repair and replication, BER. DEGs in the UVB‐SIPS+ALA‐PDT group showed sustained activation and higher expression levels compared to those in the UVB‐SIPS group. Cluster 3 was mainly involved in negative regulation of cell proliferation, wound healing, positive regulation of RNA polymerase II promoter transcription and negative regulation of apoptosis. Cluster 4 was mainly related with positive regulation of neuronal death, mitochondrial function, and SMAD protein signalling transduction. The DEGs in both clusters were highly expressed in the UVB‐SIPS group, but expression decreased after low‐dose ALA‐PDT treatment. Cluster 5 was enriched in GO terms related to positive regulation of transcription, positive regulation of stress fibre assembly, and osteoblast differentiation. Cluster 6 involved GO terms associated with regulation of cell apoptosis processes, assembly of macromolecular complexes, and cell adhesion. The DEGs in these two clusters were downregulated in the UVB‐SIPS group but upregulated after low‐dose ALA‐PDT treatment.

As shown in the KEGG enrichment analysis (Figure 5B), Clusters 1, 3 and 4 were enriched in the TGF‐β signalling pathway, mTOR signalling pathway, PI3K‐Akt signalling pathway, autophagy, various metabolic processes (steroid, protein synthesis, and cholesterol metabolism), and neurodegenerative pathways (Parkinson's disease, Alzheimer's disease, and amyotrophic lateral sclerosis). Cluster 2 was associated with BER pathway, chemokine signalling pathway, and DNA replication. Cluster 5 was related to cancer pathways.

### 3.6 Key DEGs in the BER pathway regulated by low‐dose ALA‐PDT

**FIGURE 6:**
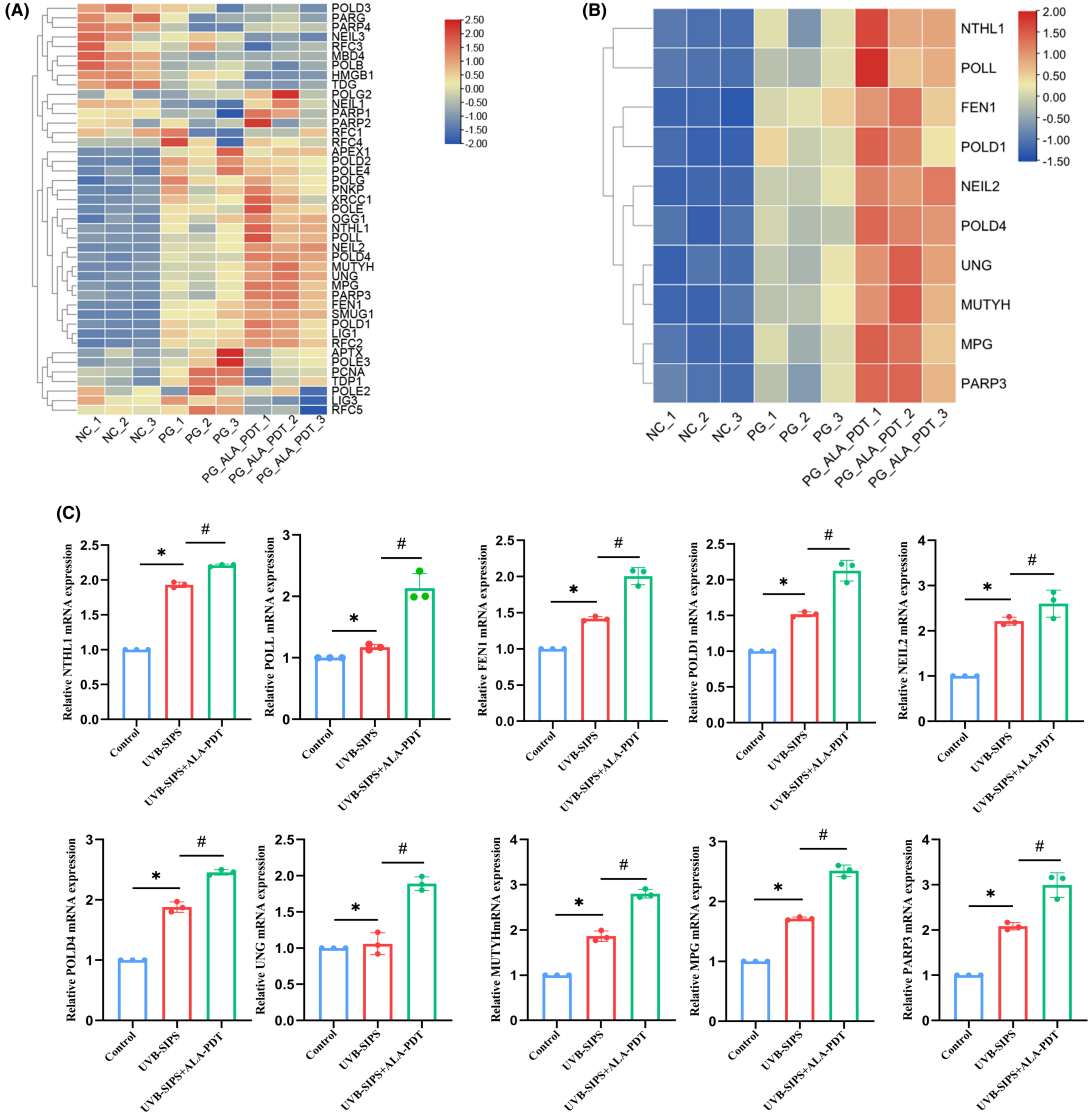
Screening and validation of key genes in the BER pathway involved in ALA‐PDT effects. (A) Heatmap of BER pathway‐related genes confirmed using the KEGG database. (B) Heatmap of DEGs in the BER pathway identified in GO enrichment analysis. (C) mRNA expression levels of DEGs were detected by qRT‐PCR. *p < 0.05 vs. Control group, #*p* < 0.05 vs. UVB‐SIPS group, *N* = 3.

Forty‐three genes implicated in the BER pathway were identified from the KEGG Database. Heatmaps showed their significant differences in expression between the groups (Figure 6A). Subsequently, we selected 10 key upregulated genes, including NTHL1, POLL, FEN1, POLD1, NEIL2, POLD4, UNG, MUTYH, MPG and PARP3, from the David database (Figure 6B). The mRNA expression levels of these 10 key genes were validated using qRT‐PCR, and their upregulations were consistent with those observed in our RNA‐seq analysis (Figure 6C).

### 3.7 Low‐dose ALA‐PDT activated the BER signalling pathway to attenuate photoaging and DNA damage

**FIGURE 7:**
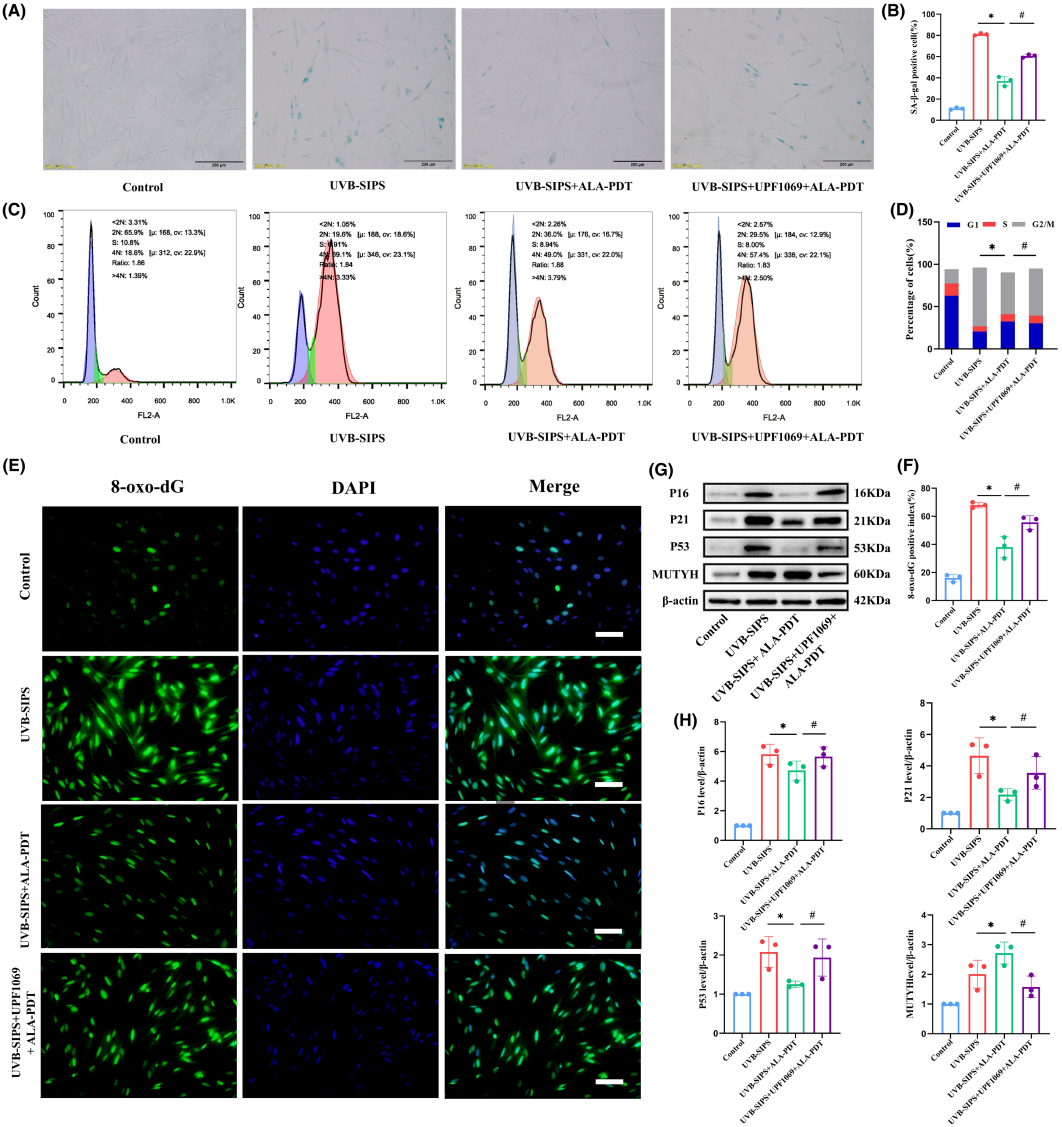
Effect of BER pathway inhibition on low‐dose ALA‐PDT to ameliorate photoaged HDFs. Photodamaged cell models were co‐treated with BER inhibitor (UPF1069) and low‐dose ALA‐PDT. (A) SA‐β‐gal staining. Bar = 200 μm, N = 3. (B) Quantitative analysis of SA‐β‐gal staining positive cells. (C) Cell cycle analysis of HDFs in each group was performed using flow cytometry. (D) The percentage of cells in each phase of the cell cycle. (E) The detection of 8‐oxo‐dG‐positive cells in each group of HDFs by immunofluorescence assay. Bar = 400 μm, *N* = 3. (F) Quantitative analysis of 8‐oxo‐dG‐positive cells in each group of cells. (G) The levels of P16, P21, P53 and MUTYH proteins in each group of HDFs were detected by Western blot. (H) The bar graph represents the ratio of the grey values of the target protein/β‐Actin. *p < 0.05 vs. UVB‐SIPS group, #*p* < 0.05 vs. UVB‐SIPS+ALA‐PDT group.

To determine whether low‐dose ALA‐PDT activates the BER signalling pathway to attenuate photoaging and DNA damage, we co‐treated photoaged HDFs with low‐dose ALA‐PDT using UPF1069 (an inhibitor of BER). As shown in Figure 7A,B, the percentage of SA‐β‐gal‐positive cells in the UVB‐SIPS+UPF1069 + ALA‐PDT group was 63.90 ± 1.31%, significantly higher than the 39.30 ± 0.80% in the UVB‐SIPS+ALA‐PDT group (*p* < 0.05). Additionally, flow cytometry analysis revealed that the G2/M phase cell population in the UVB‐SIPS+ALA‐PDT group was 49.33 ± 1.04%, but increased to 55.50 ± 1.65% after co‐treatment with UPF1069 and low‐dose ALA‐PDT in photoaged HDFs (*p* < 0.05) (Figure 7C,D). Moreover, compared to those in the UVB‐SIPS+ALA‐PDT group, the expression of aging‐related proteins P16, P21, P53 was significantly increased and that of MUTYH, a key protein of the BER pathway, was significantly decreased in the UVB‐SIPS+UPF1069 + ALA‐PDT group (*p* < 0.05) (Figure 7G,H). Finally, immunofluorescence results (Figure 7E,F) showed that the percentage of 8‐oxo‐dG‐positive cells in the UVB‐SIPS+ALA‐PDT group was 38.0 ± 7.55%, but increased to 55.67 ± 4.93% in the UVB‐SIPS+UPF1069 + ALA‐PDT group (*p* < 0.05).

## 4 DISCUSSION

The findings of this study demonstrated that low‐dose ALA‐PDT activated the BER signalling pathway, thereby enhancing DNA repair capacity, accelerating the clearance of the DNA photoproduct 8‐oxo‐dG and subsequently alleviating DNA damage. This mechanism contributes to the therapeutic effects of low‐dose ALA‐PDT against photoaging.

In this study, we found that low‐dose ALA‐PDT countered UVB‐induced skin photoaging both in vivo and in vitro. Wang, P et al. established a mouse model of photoaging induced with combined UVA and UVB radiation, showing that low‐dose ALA‐PDT significantly curbed epidermal thickening, collagen down‐regulation, and wrinkle formation in the photoaging mice.^13^ Here, we used UVB irradiation to induce photoaging in a rat model, in which ALA‐PDT achieved similar effects on overall appearance, histomorphology and collagen fibres of the skin. Additionally, we observed a reduction in elastic fibre degeneration. Another study showed that ALA‐PDT could reduce SA‐β‐gal activity and the expression levels of cellular senescence‐related proteins P16 and P21 in HDFs photoaged by UVA radiation,^4^ which is consistent with the results of our cellular experiments. Therefore, the UVB‐induced photoaging model in this study is suitable for evaluating the therapeutic effects of ALA‐PDT.

In our study, RNA‐seq analysis found that the action mechanism of low‐dose ALA‐PDT was multifaceted, primarily involving cellular autophagy and apoptosis, and its main pathways were mTOR, TGF‐β, PI3K‐Akt signalling pathways. Additionally, low‐dose ALA‐PDT enhances DNA damage repair, mainly through genes related to the BER pathway. Previous studies have investigated the regulatory effects of ALA‐PDT on cellular autophagy, apoptosis, and related signalling pathways. It was reported that ALA‐PDT promoted apoptosis and autophagy in cells by targeting the PI3K/AKT/mTOR signalling pathway.^14^ Moreover, the PI3K/Akt signalling pathway also elicited the effect of ALA‐PDT on UV‐induced skin squamous cell carcinoma in mouse models.^15^ Another study also demonstrated that ALA‐PDT improved the appearance of photoaging by remodelling collagen protein through activating the TGF‐β signalling pathway.^13^ However, the role of the BER pathway in ALA‐PDT treatment is still unclear.

The BER pathway is the major tinker for repairing DNA damage caused by oxidative stress, including UV radiation.^16^, ^17^ Research studies indicated BER activity impairment and oxidative DNA damage during the aging process.^18^ The defects in the BER repair pathway could lead to the development of premature aging phenotypes.^19^ Here, through bioinformatics analysis, we identified 10 up‐regulated genes associated with the BER pathway after low‐dose ALA‐PDT treatment, including NTHL1, POLL, FEN1, POLD1, NEIL2, POLD4, UNG, MUTYH, MPG and PARP3. FEN1 is an endonuclease primarily responsible for the excision of DNA and RNA substrates during the BER process, and the activation of various DNA metabolic pathways.^20^ MUTYH has been confirmed to protect cells from UV‐induced DNA damage.^21^ Previous studies demonstrated that when the BER pathway was initiated, MUTYH recognized and removed 8‐oxo‐dG and its adenine pairing.^22^, ^23^ MPG and UNG, as two glycosylases, are also closely engaged in the BER pathway.^24^, ^25^ Based on these findings, we speculate that the mechanism by which ALA‐PDT regulates DNA damage repair is associated with the BER pathway and involved genes.

Numerous studies have shown that DNA damage plays a crucial role in the mechanism of UV‐induced photoaging.^26^, ^27^ Firstly, DNA damage triggers mitochondrial dysfunction.^28^ Secondly, UV‐induced DNA damage causes functional degeneration and depletion of stem cells.^7^ Thirdly, UV‐induced DNA damage products, such as 8‐oxo‐dG,^29^ if not cleared in time by the native DNA‐repairing mechanisms, can further exacerbate skin photoaging and even contribute to the development of skin cancer.^30^ Wang et al. reported that recombinant photolyase‐thymidine protein effectively repaired CPDs photoproducts and reduced DNA damage to inhibit photoaging.^31^ The level of ROS generated by ALA‐PDT varies with its dosage, leading to significant different effects on DNA damage. Abo‐Zeid et al. demonstrated that high‐dose ALA‐PDT induced DNA damage in breast adenocarcinoma (MCF‐7) and hepatocellular carcinoma (HepG2) cells.^32^ This effect may imply a mechanism in which high‐dose ALA‐PDT produces high‐level ROS and subsequent severe mitochondrial damage, thus arousing cell apoptosis and/or necrosis. However, another study found that low‐dose ALA‐PDT could repair DNA damage in UVA‐induced skin photoaging, possibly by upregulating the Bach2 protein through low levels of ROS.^4^ The present study also provided the first evidence that low‐dose ALA‐PDT can repair DNA damaged by UVB. Therefore, it is speculated that low‐dose ALA‐PDT can help cells cope with oxidative stress and enhance DNA repair to impede photoaging.

Accumulating evidence suggests that the DNA‐repairing capacity can be enhanced to realize an anti‐photoaging effect.^27^, ^33^, ^34^ Chen et al. revealed that metformin reduced DNA damage and protected cells from UVA‐induced photoaging by activating the PI3K/AKT/mTOR pathway.^35^ Gao et al. found that exosomes from adipose‐derived stem cells (ADSCs) reduced UVB‐induced DNA damage by activating the Nrf2 pathway.^36^ In addition, Hwang et al. demonstrated that conditioned medium of neural stem cells (NSC‐CM), by means of its secreted protein TIMPs, modulated the NF‐κβ pathway and DNA‐repairing enzyme Rad50 to inhibit DNA damage and UVB‐induced skin photoaging.^37^ Recent studies also suggested that the overexpression of δ‐catenin in keratinocytes induced DNA‐repairing responses through the activation of the RSK2/YB1 signalling pathway.^38^ The above studies indicate that DNA damage repair counteracts with photoaging induced by UV radiation through multiple pathways. Kim et al. observed that red light enhanced the removal of oxidative DNA by activating the BER signalling pathway, thereby protecting against UV radiation.^17^ Consistently, in our study, we observed that a BER inhibitor significantly relieved photoaging manifestations after low‐dose ALA‐PDT treatment, suggesting that the BER pathway may play a crucial role in the protective mechanisms of low‐dose ALA‐PDT against photoaging.

However, there are some shortcomings in this experiment. Although we demonstrated the protective effects of low‐dose ALA‐PDT on UVB‐induced photoaging through in vitro and in vivo experiments, further validation is needed to characterize the mechanism of the BER pathway and its efficacy in vivo. Additionally, more investigation is required to determine if other regulatory pathways are involved in the effect of low‐dose ALA‐PDT.

## 5 CONCLUSION

In this study, we have found that low‐dose ALA‐PDT enhances the DNA‐repairing capacity of HDFs to facilitate the clearance of the photoproduct 8‐oxo‐dG and protect skin cells from UVB‐induced DNA damage. This protective mechanism involves the BER pathway. This study provides a theoretical basis for low‐dose ALA‐PDT in the treatment of photoaging.

## AUTHOR CONTRIBUTIONS

**Jing Wang:** Conceptualization (equal); data curation (equal); writing – original draft (equal). **Li Gu:** Conceptualization (equal); data curation (equal); writing – original draft (equal). **Zhinan Shi:** Conceptualization (equal); data curation (equal); writing – original draft (equal). **Zhiyi Xu:** Resources (equal); software (equal). **Xiaoyu Zhai:** Resources (equal); software (equal). **Shu Zhou:** Resources (equal); software (equal). **Jingting Zhao:** Resources (equal); software (equal). **Liqun Gu:** Investigation (equal). **Lin Chen:** Methodology (equal). **Linling Ju:** Methodology (equal). **Bingrong Zhou:** Conceptualization (equal); writing – review and editing (equal). **Hui Hua:** Conceptualization (equal); project administration (equal); writing – review and editing (equal).

## FUNDING INFORMATION

This work was supported by the National Natural Science Foundation of China (82073472), Postgraduate Research & Practice Innovation Program of Jiangsu Province (SJCX23_1804), the Nantong Science Foundation (MS22022102), the Nantong Clinical Key Discipline Program of Integrative Medicine, and the Nantong Young Medical Key Talent Program.

## CONFLICT OF INTEREST STATEMENT

The authors declare no conflict of interest in the publication of this article.

## Data Availability

RNA‐seq data have been stored in the NCBI repository (PRJNA1048364) and will be available. Additional data supporting the results of this study are available upon reasonable request from the corresponding author.
